# Management of maternal anaphylaxis in pregnancy: a case report

**DOI:** 10.1002/ams2.238

**Published:** 2016-11-10

**Authors:** Yasunobu Tsuzuki, Mitsuo Narita, Masayuki Nawa, Urara Nakagawa, Toshiaki Wakai

**Affiliations:** ^1^ Department of Pediatrics and Allergy Sapporo Tokushukai Hospital Sapporo Hokkaido Japan; ^2^ Department of Pediatrics Sapporo Tokushukai Hospital Sapporo Hokkaido Japan; ^3^ Department of Obstetrics and Gynecology Sapporo Tokushukai Hospital Sapporo Hokkaido Japan; ^4^ Department of General Medicine Sapporo Tokushukai Hospital Sapporo Hokkaido Japan

**Keywords:** Anaphylactic shock, fetal brain damage, fetal distress, food allergy

## Abstract

**Case:**

A 26‐year‐old woman (gravida 2, para 1) at 25 weeks’ gestation was brought to the emergency department because of anaphylactic symptoms. She reported eating Japanese soba and developed symptoms of dyspnea, generalized itchy rash, abdominal pain, and severe uterine contractions within 15–30 min of eating. She was immediately treated by normal saline infusion, two injections of epinephrine (intramuscularly), and a nebulized short‐acting β_2_‐receptor agonist, followed by H_1_‐antihistamine and methylprednisolone. Obstetrical management was undertaken by an obstetrician.

**Outcome:**

The patient recovered rapidly without a biphasic reaction of anaphylaxis. After 11 weeks, a healthy, neurologically intact baby was born.

**Conclusion:**

Management of anaphylaxis in pregnant patients is basically the same of that in non‐pregnant ones. Treatment should commence immediately to prevent further development of the anaphylaxis reaction and fetal neurological deficiency.

## Introduction

Anaphylaxis is a rare event during pregnancy, but its onset may trigger maternal hypotension leading to intrapartum asphyxia and, eventually, the risk of severe fetal brain damage. Furthermore, the risk of cesarean delivery in anaphylactic patients in pregnancy is high (74%).^1^ We report a case of maternal anaphylaxis that was caused by food allergy and discuss the management of anaphylaxis in a pregnant patient.

## Case

**Figure 1:**
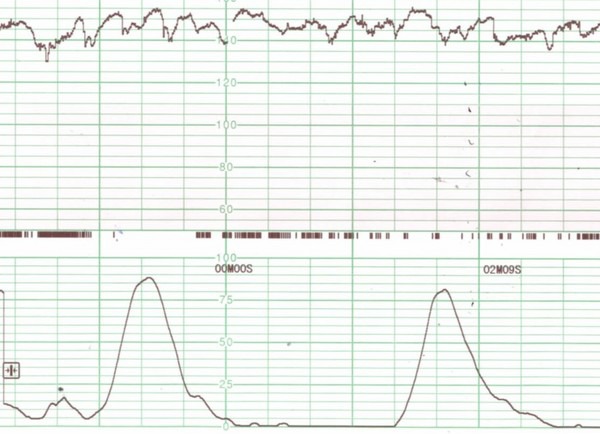
Fetal heart rate during maternal anaphylaxis. The mother experienced strong and frequent uterine contractions (lower panel), but examination of the fetus revealed a normal heart rate (140–160 b.p.m) with variability (upper panel).

A 26‐year‐old woman (gravida 2, para 1) at 25 weeks’ gestation was brought to the emergency department because of severe uterine contractions, dyspnea, generalized itchy rash, abdominal pain, and peripheral cyanosis. She reported eating buckwheat noodles (soba) and tempura (fish, shrimp, crab, and vegetables) and developed the symptoms within 15–30 min of eating. She had a medical history of atopic dermatitis, but no history of food allergies or anaphylaxis. She was lucid. On examination, her blood pressure was 104/72 mmHg, heart rate was 112 b.p.m., respiratory rate was 40 breaths/min, oxygen saturation was 100% (with high‐flow humidified supplemental 100% oxygen 6 L/min by mask), and body weight was 43 kg. The patient was diagnosed with anaphylaxis because of a suspected food allergy, and she was immediately treated with normal saline infusion, an i.m. injection of 0.4 mg epinephrine, and a nebulized short‐acting β_2_‐receptor agonist. We manually displaced her uterus to the left to prevent inferior vena cava compression. The treatment was effective, but her generalized itchy rash and abdominal pain persisted. She received an additional 0.4 mg epinephrine i.m. as well as an H_1_‐antihistamine and methylprednisolone. Serum allergen‐specific immunoglobulin E (IgE) testing (total IgE 360 IU/mL, dust mite 86 UA/mL, buckwheat 0.1 UA/mL, wheat 1.79 UA/mL) was carried out in the emergency department; total serum tryptase was increased on day 1 (15.5 μg/mL) but decreased by day 2 (3.9 μg/mL). Obstetrical management was undertaken by an obstetrician for threatened preterm labor, but the fetal heart rate was almost normal (140–160 beats/min) with variability (Fig. 1), and ultrasonography showed a vital fetus with no fetal distress. A cesarean delivery was not undertaken. After the second injection of epinephrine, the patient's condition stabilized and both the mother and fetus rapidly recovered. A biphasic reaction of anaphylaxis did not occur. However, the patient required fetal monitoring and maternal treatment of threatened preterm labor for several days. After the seventh hospital day, the patient was discharged. Eleven weeks later, the baby was born without neurological deficiency.

The cause of the allergy was investigated after birth, because skin prick and challenge test are contraindicated in pregnancy considering the risk of inducing anaphylaxis. The skin prick test was positive for dust mite (4.4 mm × 1.1 mm), buckwheat (3.7 mm × 4.2 mm), and wheat (4.7 mm × 3 mm) and was negative for shrimp (0 mm). An oral food challenge for wheat and buckwheat revealed the anaphylaxis was caused by a buckwheat allergy. She eliminated buckwheat from her diet and has experienced no further allergy symptoms.

## Discussion

Anaphylaxis is defined as a serious, generalized or systemic, allergic or hypersensitivity reaction that can be life‐threatening or fatal.^2^ Anaphylaxis is a relatively infrequent event during pregnancy.^2^, ^3^, ^4^, ^5^ Management of anaphylaxis in pregnant patients is basically the same as that in non‐pregnant ones.^3^, ^4^, ^5^, ^6^ Symptoms of anaphylaxis include respiratory (e.g., wheeze, dyspnea), gastrointestinal (e.g., vomiting, abdominal pain), skin and mucosal (e.g., urticaria, itchy rash, swelling of lips), and cardiovascular and central nervous systems (e.g., reduced blood pressure, feeling faint, headache).^7^ Potential pregnancy‐related symptoms and signs of anaphylaxis are lower back pain, fetal distress, uterine cramps, preterm labor, and vulvar or vaginal itching.^3^ Anaphylaxis may also trigger maternal hypotension and hypoxemia. Maternal hypoxemia and hypotension are potentially life‐threatening to both mother and fetus. Maternal hypoxemia can lead to intrapartum asphyxia, and maternal hypotension and vasoconstriction can result in decreased uterine blood flow.^3^, ^4^, ^5^, ^6^ Risks to the fetus include hypoxic–ischemic encephalopathy, severe central nervous system damage, or death.^3^, ^4^, ^5^, ^6^ Initial treatment includes improving maternal airway, breathing, and circulation, removing causative agents, and injecting 0.01 mg/kg epinephrine in the mid‐outer thigh.^3^ The initial dosing is based on recommendations of several allergy organizations, particularly, epinephrine (0.01 mg/kg of a 1:1000, 1 mg/mL solution) i.m. injection to a maximum of 0.5 mg in adults and 0.3 mg in children.^2^, ^7^, ^8^ It is important to re‐evaluate the allergic symptoms of the patient, to prevent permanent damage to the fetus. If allergy symptoms recur or do not improve, epinephrine injection can be needed repeatedly every 5–15 min.^2^, ^3^, ^7^ Therefore, our patient was immediately treated with normal saline infusion, supplemental 100% oxygen, and two i.m. injections of epinephrine. Patients may require cesarean delivery to avoid fetal hypoxemia and prevent severe fetus damage. In cases of anaphylaxis during pregnancy, both mother and fetus must be treated. Therefore, the patient must be seen by a specialist team comprising obstetrics, neonatology, and anesthesiology for medical and surgical obstetric management, such as fetal monitoring, maternal treatment of threatened preterm labor, and preparing cesarean delivery.^3^ The epinephrine auto‐injector should be prepared for pregnant women who has potential risk of anaphylaxis.

## Conclusion

We present a case of maternal anaphylaxis caused by food allergy, with details of patient management. Allergic and obstetric management should be commenced immediately to prevent further development of the anaphylactic reaction and fetal brain damage. Drugs for anaphylaxis, such as epinephrine, antihistamines, glucocorticoids, and vasopressors, can be used safely without major side‐effects in pregnancy. Certainly, potential side‐effects of any drugs should be considered.

## Conflict of interest

None declared.

## References

[1] Mulla ZD , Ebrahim MS , Gonzalez JL . Anaphylaxis in the obstetric patient: analysis of a statewide hospital discharge database. Ann. Allergy Asthma Immunol. 2010; 104: 55–9. 20143646 10.1016/j.anai.2009.11.005

[2] Simons FER , Schatz M . Anaphylaxis during pregnancy. J. Allergy Clin. Immunol. 2012; 130: 597–606. 22871389 10.1016/j.jaci.2012.06.035

[3] Berardi A , Rossi K , Cavalleri F , *et al* Maternal anaphylaxis and fetal brain damage after intrapartum chemoprophylaxis. J. Perinat. Med. 2004; 32: 375–7. 15346827 10.1515/JPM.2004.070

[4] Chaudhuri K , Gonzales J , Jesrun CA , Ambat MT , Mandal‐Chaudhuri S . Anaphylactic shock in pregnancy: a case study and review of the literature. Int. J. Obstet. Anesth. 2009; 17: 350–7. 10.1016/j.ijoa.2008.05.002 18691872

[5] Berenguer A , Couto A , Brites V , Fernandes R . Anaphylaxis in pregnancy: a rare cause of neonatal mortality. BMJ Case Rep. 2013. doi:10.1136/bcr-2012-007055. Available from: http://www.ncbi.nlm.nih.gov/pmc/articles/PMC3603634/ 10.1136/bcr-2012-007055 PMC3603634 23314874

[6] Simons FER . Anaphylaxis. J. Allergy Clin. Immunol. 2010; 125: 161–81. 10.1016/j.jaci.2009.12.981 20176258

[7] Simons FER , Ardusso LRF , Bilò MB , *et al* World Allergy Organization anaphylaxis guidelines: summary. J. Allergy Clin. Immunol. 2011; 127: 587–93. 21377030 10.1016/j.jaci.2011.01.038

[8] Simons FER , Ardusso LRF , Bilò MB , *et al* International consensus on (ICON) anaphylaxis. World Allergy Organ. J. 2014; 7: 9 Available from: http://www.ncbi.nlm.nih.gov/pmc/articles/PMC4038846/ 24920969 10.1186/1939-4551-7-9 PMC4038846

